# Long non-coding RNA DPP10-AS1 exerts anti-tumor effects on colon cancer *via* the upregulation of ADCY1 by regulating microRNA-127-3p

**DOI:** 10.18632/aging.202729

**Published:** 2021-03-19

**Authors:** Gang Liu, Hui Zhao, Qiang Song, Guangmeng Li, Shuying Lin, Sheng Xiong

**Affiliations:** 1 Institute of Biomedicine and National Engineering Research Center of Genetic Medicine, College of Life Science and Technology, Jinan University, Guangzhou 510632, P. R. China; 2 Central Laboratory, Chongqing Three Gorges Central Hospital, Chongqing 404000, P. R. China; 3 Department of Biomedical Engineering, School of Basic Medical Sciences, Guangzhou Medical University, Guangzhou 511436, P. R. China; 4 Guangdong Province Engineering Research Center for Antibody Drug and Immunoassay, Jinan University, Guangzhou 510632, P. R. China

**Keywords:** colon cancer, cancer stem cells, long non-coding RNA DPP10-AS1, microRNA-127-3p, adenylate cyclase 1

## Abstract

Herein we hypothesized that DPP10-AS1 could affect the development of colon cancer via the interaction with miR-127-3p and adenylate cyclase 1 (ADCY1). After sorting of CD133 positive cells, sphere formation, colony formation, proliferation, invasion, migration, and apoptosis were detected to explore the involvement of DPP10-AS1 and miR-127-3p in the colon cancer stem cell (CCSC) properties through gain- and loss-of function approaches. Furthermore, tumor xenograft in nude mice was conducted to investigate the effect of DPP10-AS1 and miR-127-3p on tumor growth *in vivo*. Poorly expressed DPP10-AS1 and ADCY1, while highly expressed miR-127-3p were found in CCSCs. Low expression of DPP10-AS1 was correlated with TNM stage, lymphatic node metastasis, and tumor differentiation. Upregulation of DPP10-AS1 increased ADCY1 protein expression, decreased the protein expression of CCSC-related factors, inhibited sphere formation, colony formation, proliferation, invasion and migration, and accelerated apoptosis of HT-29 and SW480 cells by suppressing the expression of miR-127-3p. Further, the above *in vitro* findings were also confirmed by *in vivo* assays. Taken together, this study demonstrates that DPP10-AS1 inhibits CCSC proliferation by regulating miR-127-3p and ADCY1, providing fresh insight into a promising novel treatment strategy for colon cancer.

## INTRODUCTION

As a disease of the digestive system, colon cancer is often accompanied by high familial transmissibility as well as a high risk of hepatic metastasis [1]. Recent studies have ranked colon cancer as the 3^rd^ most lethal cancer, wreaking havoc all across the world [2]. Risk factors associated with colon cancer include several dietary factors such as folate, alcohol and methionine [3]. Colon cancer exhibits considerable disease variability, with its heterogeneity often hindering the process of determining patients that may benefit most from adjuvant therapy as well as impeding the development of novel targeted agents [4]. More importantly, colon cancer consists of a small number of cancer stem cells which regulate tumor maintenance and induce resistance to cancer therapies, paving the way for tumor recapitulation upon the termination of treatment [5]. Therefore, it is imperative to find better and more effective treatment regimens for colon cancer based on its cancer stem cells.

As regulatory noncoding RNAs, long non-coding RNAs (lncRNAs) have been reported to play crucial roles in multiple biological processes, with their dysregulation implicated in a variety of complicated diseases [6]. At present, lncRNAs have also been highlighted to exhibit prognostic roles in patients with colon cancer [7]. Initial bioinformatics prediction analysis in the current study found that DPP10-AS1, a poorly studied lncRNA, is exhibited at low levels in colon cancer. A current study analyzed the expression profile of lncRNAs including DPP10-AS1 in breast cancer and provide useful information for exploring candidate therapeutic targets and new molecular biomarkers for HER-2-enriched subtype breast cancer [8] represent noncoding RNAs capable of regulating the expression of target mRNAs and are mediated by both tumor suppressors and promoters [9]. To the best of our knowledge, the variations in miR expression are a common characteristic in colon cancer [10]. In addition, the results of our bioinformatics prediction analysis indicated that microRNA-127-3p (miR-127-3p) was a target gene of DPP10-AS1, and further suggested to regulate the adenylate cyclase (ADCY1) gene. As a specific gene for neuronal tissues, ADCY1 is a member of the enzyme families capable of catalyzing the formation of cyclic AMP [11]. To date, there are only a handful of studies that have explored its role in colon cancer. LncRNAs have also been reported to modulate the function of miRs via their action as endogenous sponges to control gene expression, whereas miRs have been demonstrated to bind and regulate the stability of lncRNAs [12]. Based on the aforementioned findings, we hypothesized that DPP10-AS1 may function as a tumor suppressor in colon cancer by regulating miR-127-3p and ADCY1. Therefore, the current study set out elucidate the regulatory role of DPP10-AS1 in colon cancer stem cells (CCSCs) via the interaction with miR-127-3p and ADCY1, with the hope to find a better effective therapy method for colon cancer.

## RESULTS

### DPP10-AS1 regulates the expression of ADCY1 by binding to miR-127-3p in colon cancer

**Figure 1:**
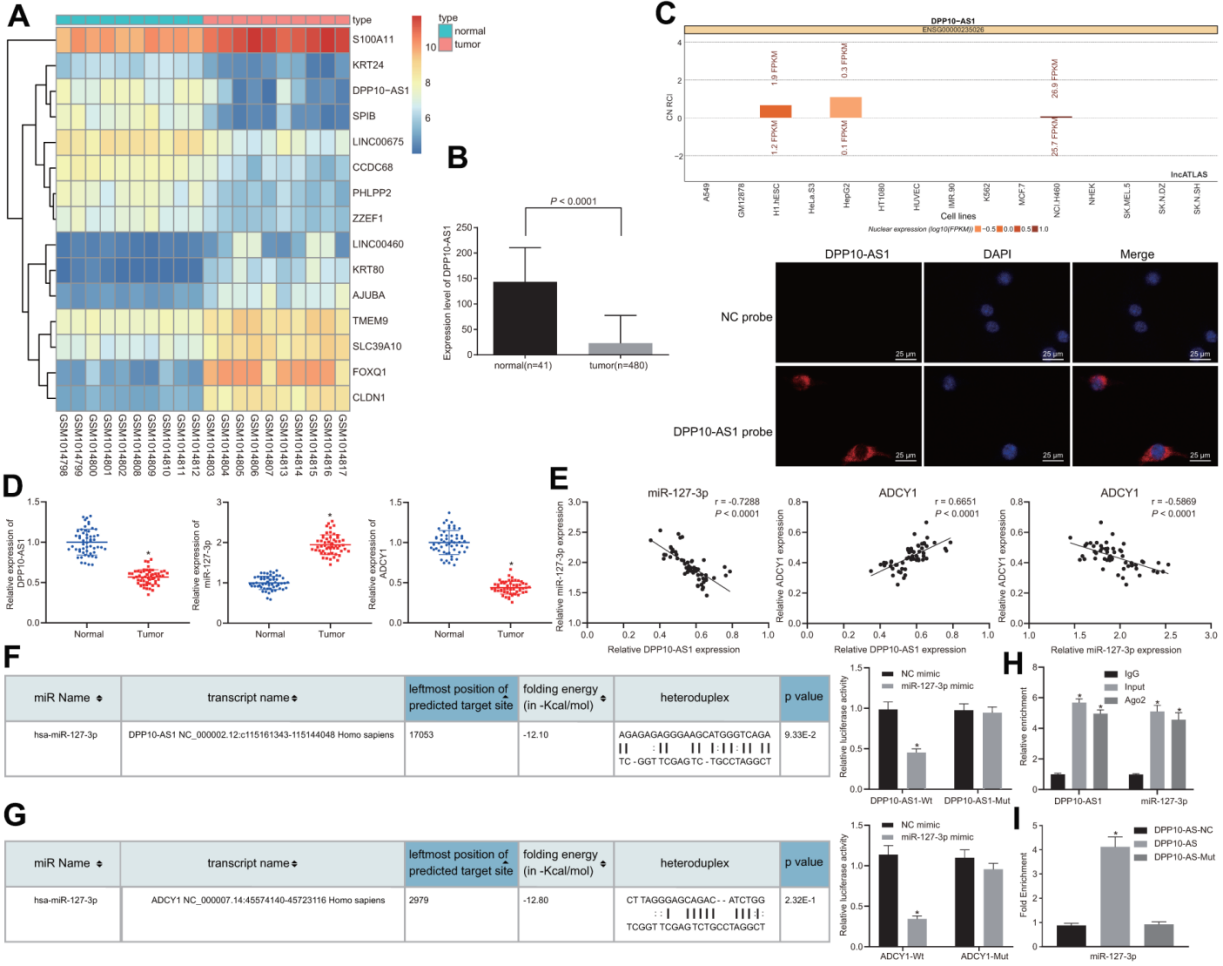
**Prediction and verification of the binding relationship among DPP10-AS1, ADCY1, and miR-127-3p.** (**A**) the heat map of microarray data GSE41328. (**B**) the expression of DPP10-AS1 in colon cancer tissues and adjacent normal tissues in TCGA database. * *p <* 0.05 compared with adjacent normal tissues. (**C**) the location of DPP10-AS1 in cells by website prediction and FISH assay. (**D**) the expression of DPP10-AS1, miR-127-3p, and ADCY1 in cancer and adjacent normal tissues detected by RT-qPCR. * *p* < 0.05 compared with adjacent normal tissues. (**E**) Pearson’s correlation coefficient on the correlation among DPP10-AS1, miR-127-3p, and ADCY1 in colon cancer. (**F**) binding relation between DPP10-AS1 and miR-127-3p predicted on a bioinformatics website and dual luciferase reporter gene assay. * *p* < 0.05 compared with NC mimic treatment. (**G**) the binding site between miR-127-3p and ADCY1 predicted using bioinformatics analysis and dual luciferase reporter gene assay. * *p* < 0.05 compared with NC mimic treatment. (**H**) interaction between DPP10-AS1 and miR-127-3p verified by RIP assay. * *p <* 0.05 compared with IgG. (**I**) enrichment of miR-127-3p in DPP10-AS1 revealed by RNA pull-down assay. *, *p* < 0.05 *vs.* DPP10-AS1 NC. Measurement data were expressed as mean ± standard deviation. The data were analyzed by *t*-test. Data among multiple groups were analyzed by one-way ANOVA followed by a Tukey’s post hoc test. The experiment was repeated three times.

Initial data analysis of the microarray dataset GSE41328 indicated that DPP10-AS1 was poorly expressed in colon cancer (Figure 1A), which was further in line with the results obtained from the TCGA database (Figure 1B). Subsequently, to further explore the specific mechanism by which DPP10-AS1 influences colon cancer, the website (http://lncatlas.crg.eu/) and FISH assay were employed in our study, the results of which revealed that DPP10-AS1 was enriched in cytoplasm (Figure 1C). The findings indicated that DPP10-AS1 could potentially bind to the downstream miRNAs to regulate genes. Thus, bioinformatics analysis was subsequently conducted, revealing the existence of specific binding sites between DPP10-AS1 and miR-127-3p. Meanwhile, binding sites between miR-127-3p and ADCY1 were also identified, suggesting that DPP10-AS1 involve in the progression of colon cancer by regulating ADCY1 through miR-127-3p. Next, RT-qPCR was conducted to determine the expression of DPP10-AS1, miR-127-3p, and ADCY1 in both the colon cancer and adjacent normal tissues. Compared with adjacent normal tissues, DPP10-AS1 and ADCY1 expression was downregulated, while miR-127-3p expression was upregulated in colon cancer tissues (Figure 1D). Moreover, based on the Pearson’s correlation coefficient, DPP10-AS1 was identified to be positively correlated with ADCY1, while both DPP10-AS1 and ADCY1 expressions were negatively correlated with miR-127-3p expression in colon cancer patients (Figure 1E). The correlation between the expression of DPP10-AS1 and the clinicopathological parameters of colon cancer patients was further analyzed by setting the average level of DPP10-AS1 (0.568) as the threshold and dividing the colon cancer patients into the groups of high expression (n = 25) and low expression (n = 29) based on DPP10-AS1 expression. The results showed that the patients with low expressions of DPP10-AS1 presented with low tumor differentiation, III-IV stage, lymphatic node metastasis, and distant metastasis, while little correlation was found between the DPP10-AS1 expression and the age, gender, and tumor size of patients (Table 1).

**Table 1:** Correlation between DPP10-AS1 expression and clinicopathological parameters of colon cancer patients.

**Clinicopathological parameters**	**n = 54**	**Expression of DPP10-AS1**		***p*-value**
		**low expression**	**high expression**	
Age				0.6509
≤ 55	32 (59.26%)	14(43.57%)	18(56.26%)	
> 55	22 (40.74%)	11(50.00%)	11(50.00%)	
Gender				0.1589
Male	29 (53.70%)	16(55.17%)	13(44.83%)	
Female	25 (46.30%)	9(36.00%)	16(64.00%)	
TNM stage				0.0152*
I-II	17 (31.48%)	12(70.59%)	5(29.41%)	
III-IV	37 (68.52%)	13(35.14%)	24(64.86%)	
Lymphatic node metastasis				0.0345*
Yes	34 (62.96%)	12(35.29%)	22(64.71%)	
No	20 (37.04%)	13(65.00%)	7(35.00%)	
Distant metastasis				0.0163*
Yes	35 (64.81%)	12(34.29%)	23(65.71%)	
No	19 (35.19%)	13(68.42%)	6(31.58%)	
Tumor differentiation				0.0338*
High	18 (33.33%)	12(66.67%)	6(33.33%)	
Low	36 (66.67%)	13(36.11%)	23(63.89%)	
Tumor size				0.5989
≤ 5	28 (51.85%)	12(42.86%)	16(57.14%)	
> 5	26 (48.15%)	13(50.00%)	13(50.00%)	

Notes: *p < 0.05 indicates statistical significance.

Dual-luciferase reporter gene assay was then performed in order to verify the binding relationship among DPP10-AS1, miR-127-3p and ADCY1, in which the specific binding sites of DPP10-AS1 and ADCY1 were mutant, obtaining the WT-DPP10-AS1/MUT-DPP10-AS1 and WT-ADCY1/MUT-ADCY1. The results displayed that the luminescent signal in the co-transfection of miR-127-3p mimic with WT-miR-127-3p/DPP10-AS1 or the co-transfection of miR-127-3p mimic with WT-miR-127-3p/ADCY1 was decreased (Figure 1F), illustrating that ADCY1 was indeed the target gene of miR-127-3p (Figure 1G). The RIP and RNA pull-down assay results revealed that compared with cells treated with IgG, those treated with Ago2 triggered an increase of DPP10-AS1 in miR-127-3p expression (Figure 1H). Moreover, based on RNA-pull down results (Figure 1I), miR-127-3p bound to more DPP10-AS1 (*p* < 0.05).

The findings suggested that DPP10-AS1 could competitively bind to miR-127-3p to regulate the expression of ADCY1, thereby participating in the development of colon cancer.

### DPP10-AS1, miR-127-3p and ADCY1 may play important roles in maintaining the properties of CCSCs

**Figure 2:**
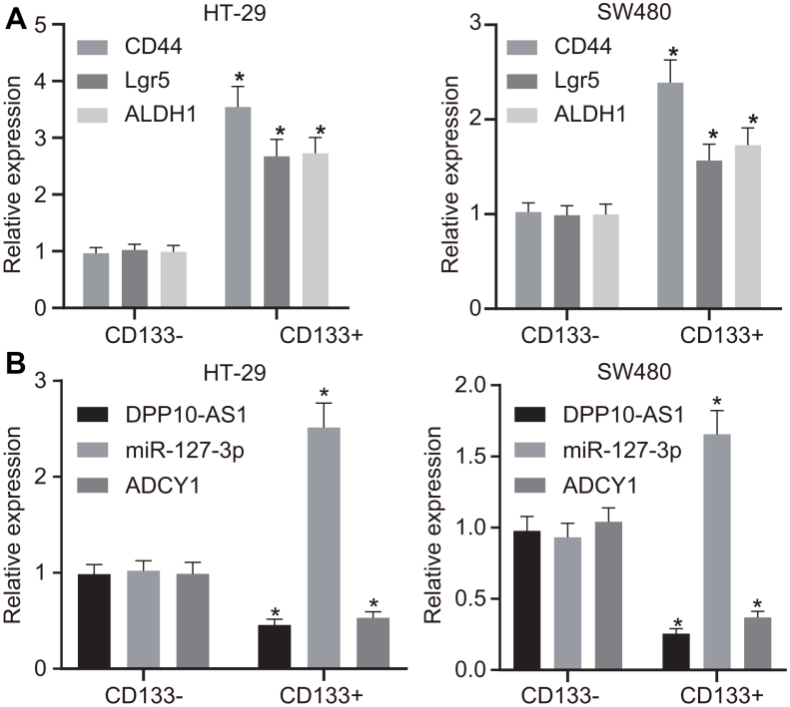
**HT-29 and SW480 cells are successfully sorted out.** (**A**) expression of stem cell markers detected by RT-qPCR. (**B**) expression of DPP10-AS1, miR-127-3p, and ADCY1 determined using RT-qPCR. Measurement data were expressed as mean ± standard deviation. The data between two groups were analyzed by unpaired *t*-test. Data among multiple groups were analyzed by one-way ANOVA followed by a Tukey’s post hoc test. The experiment was repeated three times. * *p* < 0.05 compared with CD133 negative cells.

The expression of the markers for stem cells (CD133, CD44, Lgr5, and ALDH1) was detected using RT-qPCR, and the obtained results revealed that the expression of the markers for stem cells was increased in the CD133 positive cells when compared with those in CD133 negative cells (Figure 2A). RT-qPCR was further performed to determine the expression of DPP10-AS1, miR-127-3p, and ADCY1 in CD133 positive and negative cells, which displayed that the expression of DPP10-AS1 and ADCY1 was declined, while that of miR-127-3p was enhanced in the CD133 positive cells (Figure 2B). Altogether, the results obtained suggested that DPP10-AS1, miR-127-3p, and ADCY1 may play important roles in the maintenance of CCSC properties.

### Upregulation of DPP10-AS1 suppresses expression of stem cell markers

**Figure 3:**
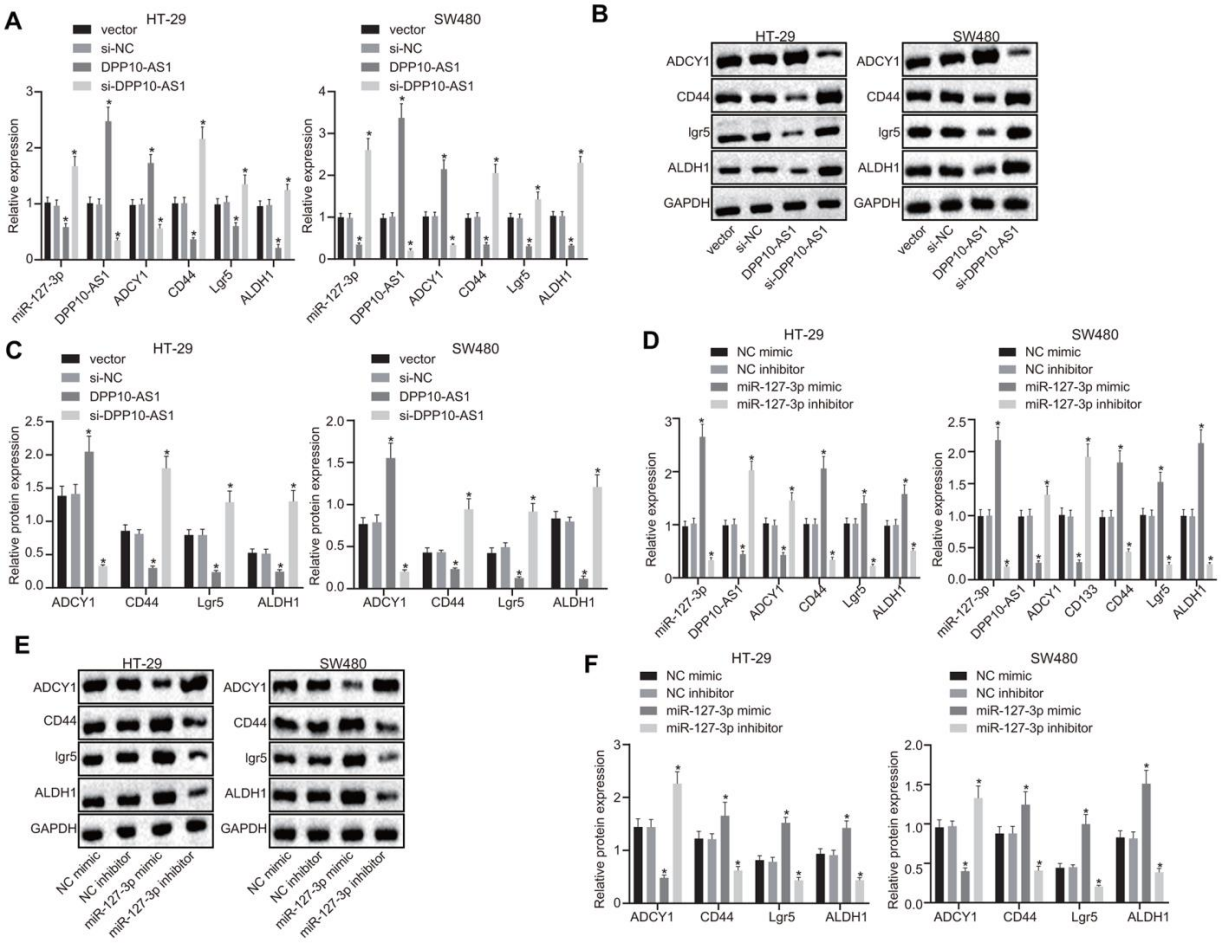
**Upregulation of DPP10-AS1 or downregulation of miR-127-3p elevates the ADCY1 protein expression and diminishes the protein expression of CD44, Lgr5, and ALDH1.** (**A**–**C**) the mRNA expression, the immunoblots and protein expression of stem cell genes determined using RT-qPCR and western blot analysis respectively after DPP10-AS1 expression was altered. (**D**–**F**) the mRNA expression, the immunoblots and protein expression of stem cell genes evaluated using RT-qPCR and western blot analysis respectively after miR-127-3p expression was altered. *, *p* < 0.05 *vs.* vector and si-NC or NC mimic and NC inhibitor. The measurement data were expressed as mean ± standard deviation. The data among multiple groups were analyzed by one-way ANOVA followed by a Tukey’s post hoc test. The experiment was repeated three times. DPP10-AS1, cells transfected with DPP10-AS1 plasmid; si-DPP10-AS1, cells transfected with si-DPP10-AS1; si-NC, cells transfected with si-negative control plasmid; miR-127-3p mimic, cells transfected with miR-127-3p mimic plasmid; NC mimic, cells transfected with negative control mimic plasmid.

Next, to investigate the effects of DPP10-AS1 and miR-127-3p on the stemness of HT-29 and SW480 stem cells, the expression of DPP10-AS1 or miR-127-3p was altered in the CD133 positive cells of the HT-29 and SW480 cells. RT-qPCR and western blot analysis results revealed that the treatment of DPP10-AS1 plasmid significantly downregulated the expression of miR-127-3p but upregulated the expression of DPP10-AS1 and ADCY1, accompanied with notable declines in the mRNA and protein levels of CD44, Lgr5, and ALDH1 (*p* < 0.05). In contrast, the si-DPP10-AS1 plasmid increased miR-127-3p expression, while decreased the expression of DPP10-AS1 and ADCY1, coupled with notable elevation in the mRNA and protein levels of CD44, Lgr5, and ALDH1 (*p* < 0.05) (Figure 3A–3C).

Furthermore, the miR-127-3p, CD44, Lgr5, and ALDH1 expression was increased, while DPP10-AS1 and ADCY1 expression was decreased significantly in the cells transfected with miR-127-3p mimic (*p* < 0.05). However, following treatment with miR-127-3p inhibitor, declined expression of miR-127-3p, CD44, Lgr5, and ALDH1 as well as increased expression of DPP10-AS1 and ADCY1 were observed (*p* < 0.05) (Figure 3D–3F). These results indicated that upregulation of DPP10-AS1 inhibited the miR-127-3p expression, ultimately promoting the expression of ADCY1, which further downregulated the expression of CD44, Lgr5, and ALDH1.

### Upregulation of DPP10-AS1 and downregulation of miR-127-3p inhibit sphere formation and colony formation of HT-29 and SW480 stem cells

**Figure 4:**
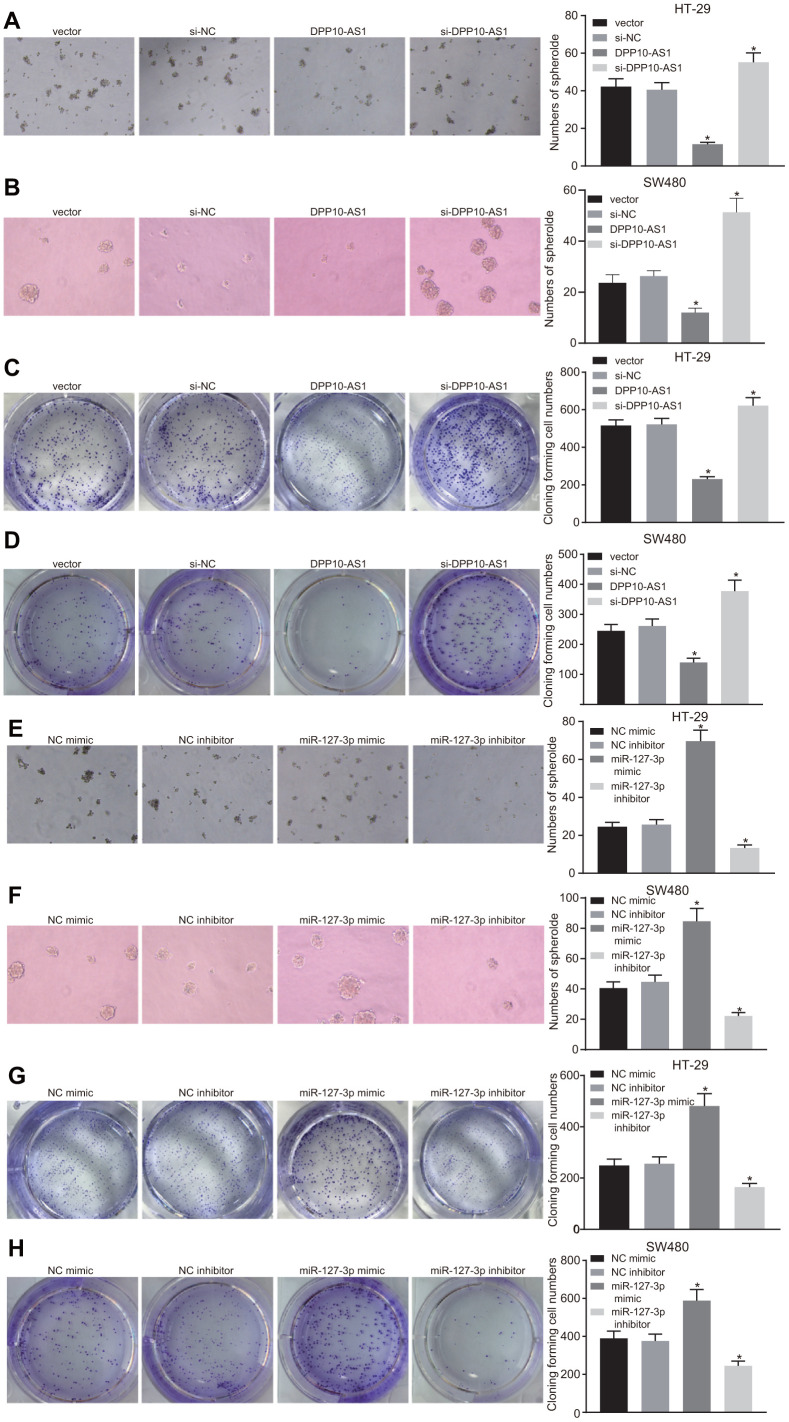
**Upregulation of DPP10-AS1 and downregulation of miR-127-3p inhibit the sphere formation and colony formation of HT-29 and SW480 stem cells.** (**A**–**D**) sphere formation and colony formation of HT29 and SW480 stem cells after DPP10-AS1 expression was altered; (**E**–**H**) sphere formation and colony formation of HT29 and SW480 stem cells after miR-127-3p expression was altered. *, *p* < 0.05 *vs.* vector and si-NC, or NC mimic and NC inhibitor. The measurement data were expressed as mean ± standard deviation. The data among multiple groups were analyzed by one-way ANOVA followed by a Tukey’s post hoc test. The experiment was repeated three times. DPP10-AS1, cells transfected with DPP10-AS1 plasmid; si-DPP10-AS1, cells transfected with si-DPP10-AS1; si-NC, cells transfected with si-negative control plasmid; miR-127-3p mimic, cells transfected with miR-127-3p mimic plasmid; NC mimic, cells transfected with negative control mimic plasmid.

Sphere formation and colony formation assays were performed to investigate the effects of DPP10-AS1 and miR-127-3p on the sphere formation and colony formation of CCSCs. In HT-29 and SW480 stem cells, DPP10-AS1 and miR-127-3p expression was altered. The results revealed that sphere formation and colony formation of HT-29 and SW480 stem cells were attenuated by DPP10-AS1, while the sphere formation and colony formation abilities were enhanced by si-DPP10-AS1 (all *p* < 0.05) (Figure 4A–4D).

In HT-29 and SW480 stem cells with varying expression of miR-127-3p, sphere and colony formation were suppressed after treatment of miR-127-3p inhibitor, while enhanced sphere formation and colony formation was detected in response to upregulation of miR-127-3p by miR-127-3p mimic (*p* < 0.05) (Figure 4E–4H). Based on the aforementioned results, it was concluded that both upregulation of DPP10-AS1 and downregulation of miR-127-3p could inhibit the sphere formation and colony formation of HT-29 and SW480 stem cells.

### Upregulation of DPP10-AS1 and downregulation of miR-127-3p suppress the proliferation but promote the apoptosis of CCSCs

**Figure 5:**
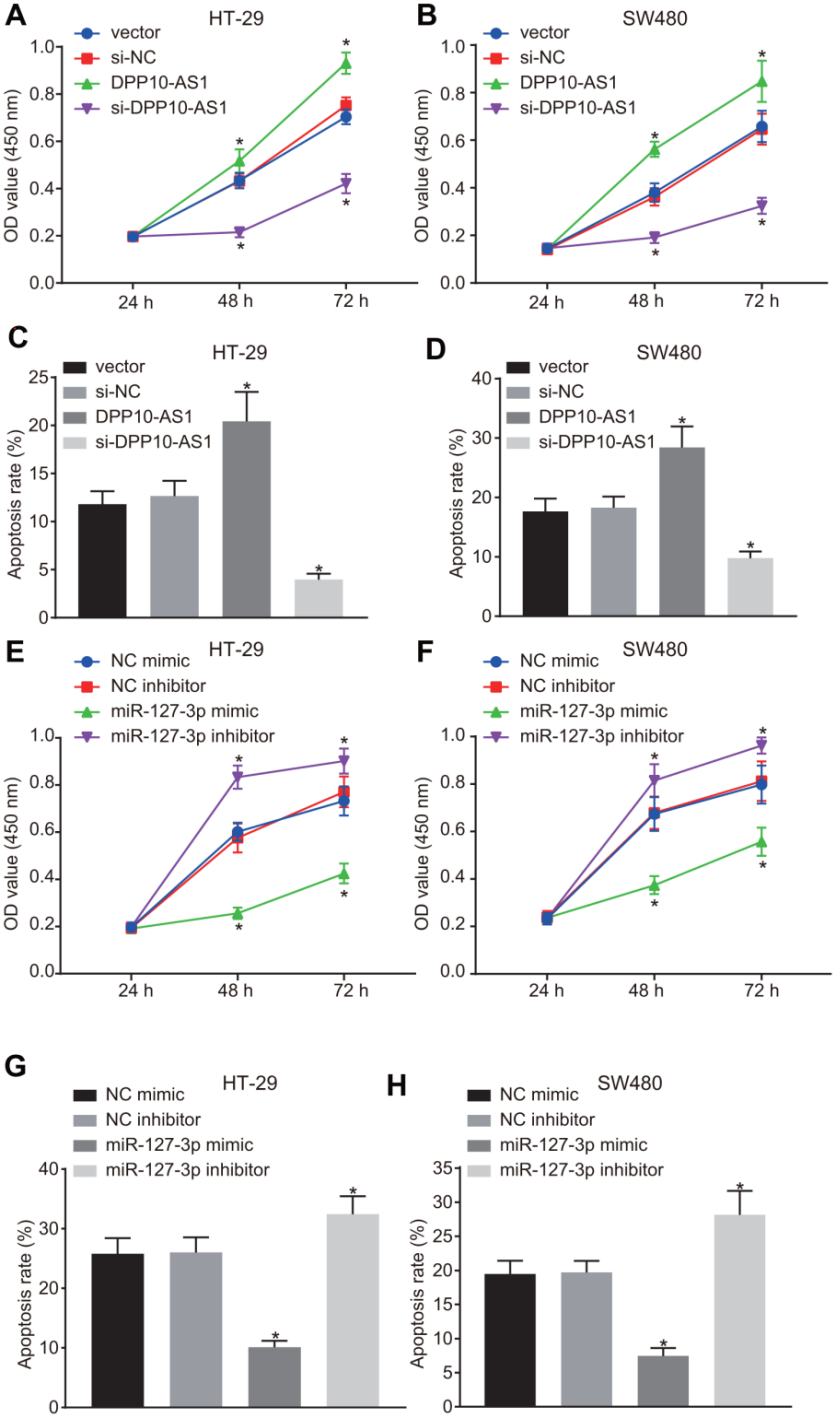
**Upregulation of DPP10-AS1 and downregulation of miR-127-3p suppress the proliferation and stimulate apoptosis of CCSCs.** (**A**, **B**) cell proliferation detected by CCK-8 assay after DPP10-AS1 expression was altered; (**C**, **D**) cell apoptosis detected by flow cytometry after DPP10-AS1 expression was altered; (**E**, **F**) cell proliferation detected by CCK-8 assay after miR-127-3p expression was altered; (**G**, **H**) cell apoptosis measured by flow cytometry after miR-127-3p expression was altered. *, *p* < 0.05 *vs.* vector and si-NC, or NC mimic and NC inhibitor. The measurement data were expressed as mean ± standard deviation. The data among multiple groups were analyzed by two-way ANOVA followed by a Tukey’s post hoc test. The experiment was repeated three times. DPP10-AS1, cells transfected with DPP10-AS1 plasmid; si-DPP10-AS1, cells transfected with si-DPP10-AS1; si-NC, cells transfected with si-negative control plasmid; miR-127-3p mimic, cells transfected with miR-127-3p mimic plasmid; NC mimic, cells transfected with negative control mimic plasmid.

The proliferation and apoptosis of HT-29 and SW480 cells with different transfection was detected by CCK-8 assay and flow cytometry. In HT-29 and SW480 stem cells with altered expression of DPP10-AS1, slower proliferation and accelerated apoptosis of HT-29 and SW480 cells were found after treatment with DPP10-AS1, while a faster proliferation and slower apoptosis were detected after treatment with si-DPP10-AS1 (all *p* < 0.05) (Figure 5A–5D).

In HT-29 and SW480 cells with altered expression of miR-127-3p, the proliferation of HT-29 and SW480 stem cells was strengthened, while the cell apoptosis was declined in cells treated with miR-127-3p mimic, yet the results were reversed after the treatment of miR-127-3p inhibitor (*p* < 0.05) (Figure 5E–5H). The above results demonstrated that upregulation of DPP10-AS1 or downregulation of miR-127-3p suppresses proliferation and promotes the apoptosis of HT-29 and SW480 stem cells.

### Upregulation of DPP10-AS1 and downregulation of miR-127-3p suppress the migration and invasion of HT-29 and SW480 stem cells

**Figure 6:**
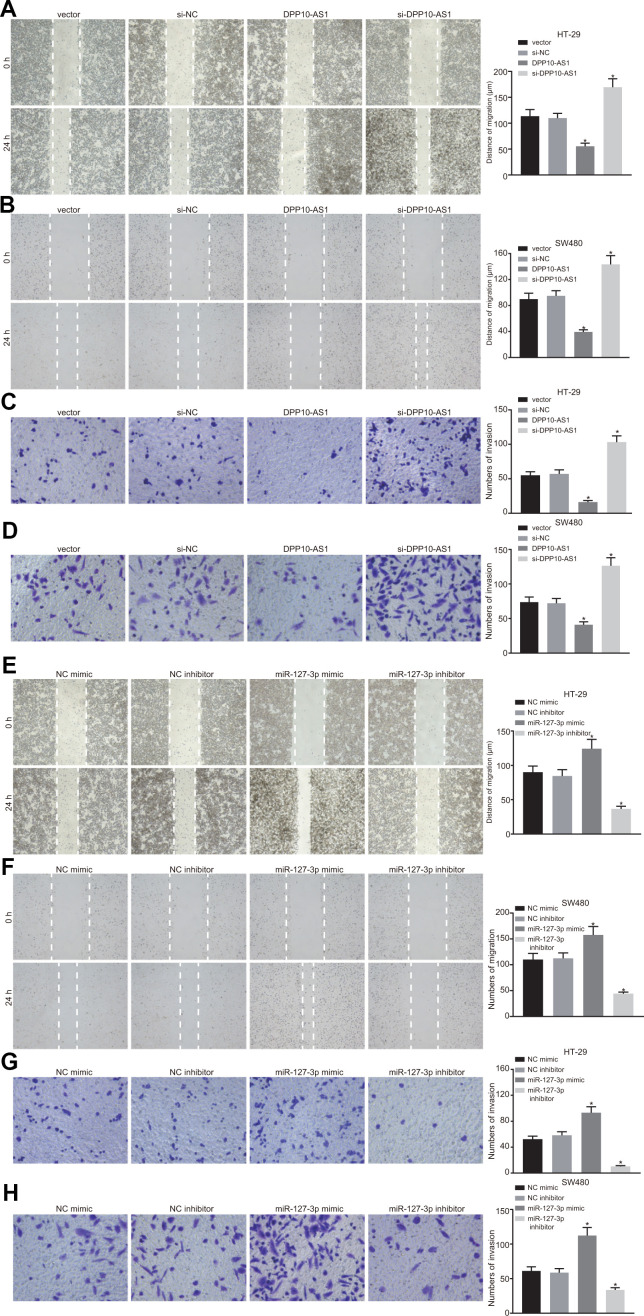
**Upregulation of DPP10-AS1 and downregulation of miR-127-3p suppress the migration and invasion of HT-29 SW480 stem cells.** (**A**–**D**) HT-29 and SW480 stem cell migration and invasion abilities evaluated by scratch test and transwell assay after DPP10-AS1 expression was altered when DPP10-AS1 was interfered. (**E**–**H**) HT-29 and SW480 stem cell migration and invasion abilities determined using scratch test and transwell assay after miR-127-3p expression was varied. *, *p* < 0.05 *vs.* vector and si-NC, or NC mimic and NC inhibitor. The measurement data were expressed as mean ± standard deviation. The data among multiple groups were analyzed by one-way ANOVA followed by a Tukey’s post hoc test. The experiment was repeated three times. DPP10-AS1, cells transfected with DPP10-AS1 plasmid; si-DPP10-AS1, cells transfected with si-DPP10-AS1; si-NC, cells transfected with si-negative control plasmid; miR-127-3p mimic, cells transfected with miR-127-3p mimic plasmid; NC mimic, cells transfected with negative control mimic plasmid.

The migration and invasion abilities of HT-29 and SW480 stem cells with different transfection were detected by scratch test and transwell assay. The results demonstrated that in HT-29 and SW480 stem cells treated with DPP10-AS1, the migration and invasion abilities decreased significantly, but after treatment with si-DPP10-AS1, the migration and invasion abilities were enhanced (all *p* < 0.05) (Figure 6A–6D).

In HT-29 and SW480 stem cells with different expression of miR-127-3p, the migration and invasion abilities of cells exhibited a notable enhancement after treatment with miR-127-3p mimic. However, the migration and invasion abilities of cells were decreased significantly by miR-127-3p inhibitor (*p* < 0.05) (Figure 6E–6H). The findings suggested that upregulation of DPP10-AS1 and downregulation of miR-127-3p suppress the migration and invasion of HT-29 and SW480 stem cells.

### Silencing of DPP10-AS1 promotes tumor growth *in vivo*

Finally, the xenograft tumor assay was performed in nude mice. At day 4 and 8, the tumor size showed no significant difference among the mice after different treatment. At day 12, a significant increase was noted.

**Figure 7:**
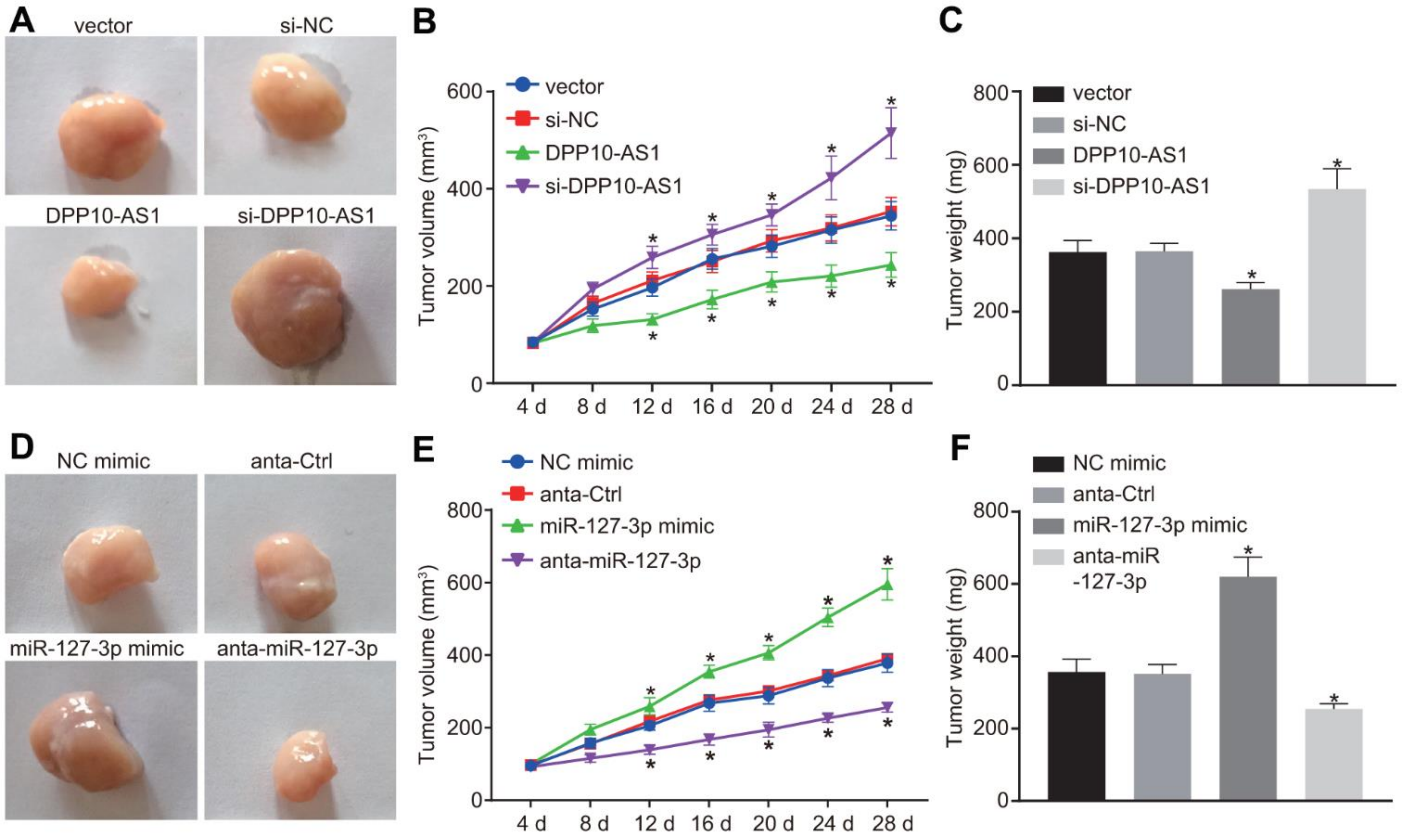
**Upregulation of DPP10-AS1 or downregulation of miR-127-3p inhibits tumor growth in HT-29 and SW480 stem cells transplanted into nude mice.** (**A**–**C**) the tumor growth and size after the expression of DPP10-AS1 was intervened. (**D**–**F**) the tumor growth and size after the expression of miR-127-3p was altered. *, *p* < 0.05 *vs.* vector and si-NC, or NC mimic and anta-Ctrl. The measurement data were expressed as mean ± standard deviation. The data among multiple groups were analyzed by one-way ANOVA followed by a Tukey’s post hoc test. The data at different time points were analyzed by repeated measures ANOVA followed by Bonferroni’s post hoc test for n = 5. The experiment was repeated three times. DPP10-AS1, cells transfected with DPP10-AS1 plasmid; si-DPP10-AS1, cells transfected with si-DPP10-AS1; si-NC, cells transfected with si-negative control plasmid; miR-127-3p mimic, cells transfected with miR-127-3p mimic plasmid; anta-miR-127-3p, cells transfected with anta-miR-127-3p plasmid; NC mimic, cells transfected with negative control mimic plasmid; anta-Ctrl, cells transfected with anta-Ctrl plasmid.

The results of tumor growth in mice with altered expression of DPP10-AS1 were shown in Figure 7A–7C. The tumor growth was hindered by DPP10-AS1 but facilitated by si-DPP10-AS1 (all *p* < 0.05). However, as the results of tumor growth in mice with different expression of miR-127-3p shown (Figure 7D–7F), the tumor growth was facilitated by miR-127-3p mimic but inhibited by anta-miR-127-3p (all *p* < 0.05). These results indicated that upregulation of DPP10-AS1 or downregulation of miR-127-3p inhibits tumor growth in nude mice.

## DISCUSSION

Colon cancer is a highly-prevalent malignancy representing a global health concern [13]. Accumulating evidences indicate that lncRNAs exert crucial functions in cancer biology, and are often aberrantly-expressed in various tumors [14]. In the current study, we set out to investigate the potential roles of DPP10-AS1 and miR-127-3p in colon cancer, with the involvement of ADCY1. Our obtained findings demonstrated that upregulation of DPP10-AS1 inhibited CCSC proliferation and stimulated apoptosis via its regulation of miR-127-3p and ADCY1 expression.

Firstly, bioinformatic prediction results in our study indicated that DPP10-AS1 and ADCY1 were poorly expressed, while miR-127-3p was highly expressed in colon cancer. The expressions of numerous lncRNAs have been found to be implicated in various types of cancers. For instance, lncRNA HOXB-AS3 was previously reported to encode a peptide to inhibit the growth of colon cancer [15]. In addition, diminished expressions of lnc-GNAT1-1 were documented in colorectal cancer, and further highlighted as a promising tumor suppressor by the virtue of RKIP-NF-κB-Snail circuit regulation [16]. Moreover, DPP10-AS1 is also known to exhibit downregulated expressions in colorectal cancer tissues, which is very much in line with our findings [17]. Meanwhile, researchers have found augmented expressions of miR-127 in cervical cancer, and also highlighted miR-127 as a potential biomarker for cervical cancer [18]. On the contrary, another study revealed tumor suppressor function of miR-127-3p in osteosarcoma [19]. Therefore, roles of miR-127-3p in different cancers remains to be further investigated. Further in accordance with our results, previous studies have recorded lowered expressions of ADCY1 in rectal adenocarcinoma metastasis, in addition to various human osteosarcomas [20, 21]. Although all these aberrant expression levels of DPP10-AS1, ADCY1 and miR-127-3p have demonstrated in other cancers, their roles in colon cancer, especially CCSC functions still remain to be elucidated, which implored us to investigate their roles in sphere formation and colony formation abilities as well as migration and invasion of colon cancer HT-29 and SW480 stem cells.

Subsequently, we uncovered that upregulation of DPP10-AS1 and downregulation of miR-127-3p brought about suppressive effects on the cellular progression and tumor growth of HT-29 cells. Evidence also suggests that another lncRNA, CASC7 quelled the proliferation as well as migration of colon cancer cells by negatively-regulating miR-21 [22]. In addition, decreased expressions of SLC25A25-AS1 have been reported to stimulate the proliferation as well as mesenchymal cell transition in colorectal cancer cells [23], which was also reflected with our findings. Meanwhile, the hard-done work of researchers has further enhanced our knowledge of miRs, with evidence revealing that miRs can exert crucial functions in a wide array of biological processes in cancer such as cell proliferation and apoptosis [24]. Further in line with our findings, the occurrence of hsa-miR-127 has also been unfounded to facilitate the proliferation, migration, and invasion of glioma cells via the regulation of replication initiator 1 [25]. More importantly, miR-127-3p, which is quickly downregulated upon differentiation, seems to be strongly stem cell-specific, and tight mediation of miR-127-3p is vital to maintain the self-renewing stem cell pool and homeostasis of the hematopoietic system [26]. In the present study, upregulation of DPP10-AS1 suppressed the cellular progression and tumor growth of CCSCs, which was achieved via its negative regulation of miR-127.

Furthermore, findings obtained demonstrated that DPP10-AS1 could inhibit the expression of miR-127-3p to upregulate ADCY1, thus contributing to the inhibition of CCSC proliferation, migration and invasion and stimulation of apoptosis. In fact, lncRNAs possess the ability to interact with the miRs as a ceRNA to regulate the expression of target genes, thus exerting vital functions in the initiation and progression of various cancers [27]. In addition, lncRNAs can also function as ceRNAs in colon cancer, and further have been suggested as implications for colon cancer prognosis [28]. In concert with the elucidated mechanism explored in our study, another lncRNA, MT1JP exerted its role as a ceRNA in the regulation of FBXW7 by competitively binding to miR-92a-3p in gastric cancer, thus inhibiting cell proliferation, migration, invasion, suppressing tumor growth and promoting cell apoptosis [29]. Moreover, MEG3 could function as a ceRNA to modulate the expressions of E-cadherin and FOXO1 by binding hsa-miR-9, thereby contributing to the repressed proliferation and invasiveness of esophageal cancer cells [30], a finding which is particularly similar to the observations made during the current study. Also, DPP10-AS1 is registered in the NCBI sequence database (NCBI reference sequence NR_036580.1) as being derived from the sequences BC032913.2 and AC066593.4. According to a recent research, BC032913 plays an inhibitory role in colon cancer aggression by upregulating TIMP3 through inactivation of the Wnt/β-catenin pathway [31]. Therefore, it would be prudent to further explore any other regulating pathways of DPP10-AS1 in colon cancer to fully utilize its therapeutic potential to reduce the burden on plagued by CCSC.

## CONCLUSIONS

**Figure 8:**
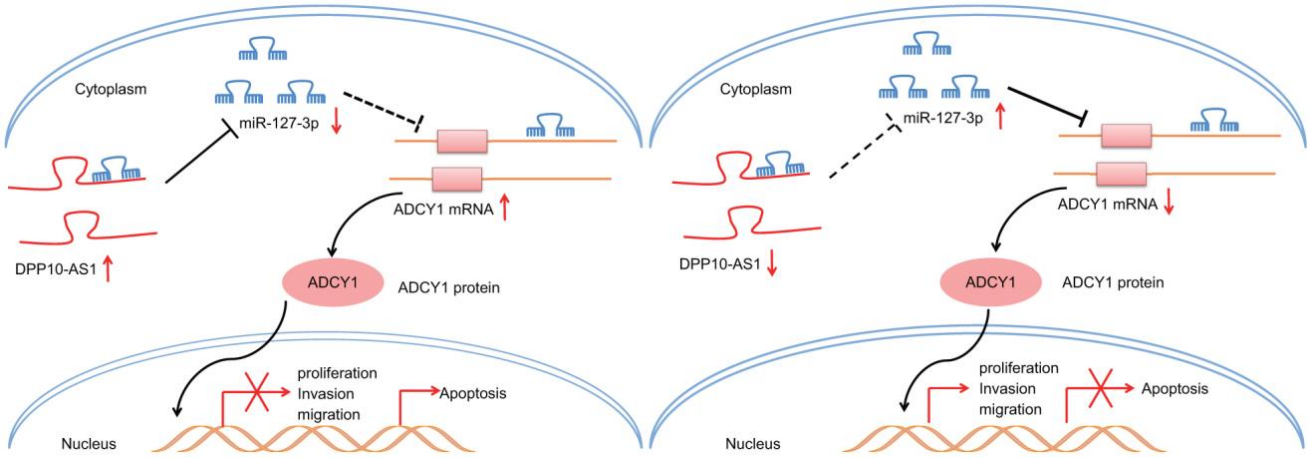
**Potential molecular mechanism of the DPP10-AS1/miR-127-3p/ADCY1 axis in CCSCs.** In CCSCs, upregulation of DPP10-AS1 inhibits the expression of miR-127-3p to increase ADCY1 expression, thereby inhibiting CCSC sphere and colony formation, proliferation, migration and invasion and promoting the apoptosis.

To conclude, upregulation of DPP10-AS1 inhibits the expression of miR-127-3p to augment ADCY1 levels, thereby inhibiting CCSC sphere and colony formation, proliferation, migration, and invasion, while promoting apoptosis (Figure 8). Our findings highlight the potential of DPP10-AS1 as a novel molecular target for the treatment of colon cancer. However, the function of lncRNA remains scantly identified. Hence, future study will be conducted in order to identify the underlying mechanisms that govern the lncRNA-miRNA interaction in our future endeavors.

## MATERIALS AND METHODS

### Ethics statement

The current study was approved by the Ethics Committee of Chongqing Three Gorges Central Hospital and performed in accordance with the principles stated in the Declaration of Helsinki. Signed informed consents were obtained from all participants prior to the study. Nude mice were used for *in vivo* studies and were cared for in accordance with the principles of the Guide for the Care and Use of Laboratory Animals published by the National Institutes of Health, with extensive efforts made to minimize the suffering of the included animals.

### Bioinformatics predication

Firstly, colon cancer-related microarray expression profiles and annotation probe files were downloaded from the Gene Expression Omnibus (GEO) database (http://www.ncbi.nlm.nih.gov/geo), and background corrected and normalized with the Affy package of R language was used for background correction and normalization [32]. The linear model-empirical Bayesian statistics method of the Limma package, combined with the conventional *t*-test, was performed to screen out the differentially expressed genes through non-specific filtration of the profile data [33]. In addition, colon cancer gene expression information was downloaded from The Cancer Genome Atlas (TCGA) database (http://cancergenome.nih.gov/), and analyzed with the R software. Differential analysis was again performed for the transcriptome profiling data with package edgeR of R [34]. False positive discovery (FDR) correction was performed on *p*-value with package multiple tests. FDR < 0.05 and log2 (fold change) >2 were set as the threshold to screen out the differentially expressed genes (DEGs).

### Clinical samples

A total of 54 colon cancer patients (29 males; 25 females; aged 34-69 years old) who underwent surgeries at Chongqing Three Gorges Central Hospital between June 2018 and May 2019 were enrolled in the current study. Based on the American Joint Committee on Cancer staging criteria, there were 17 patients at stage I-II, 37 patients at stage III-IV, 34 patients with lymphatic node metastasis, 20 patients free of lymphatic node metastasis, 35 patients with distant metastasis, 19 patients without distant metastasis, 18 patients with high tumor differentiation and 36 patients with low tumor differentiation. Patients who had received radical surgery for colon cancer and with completed clinical records were enrolled into the current study. Meanwhile, the patients with a history of chemotherapy, immunotherapy, or a combination of other malignant tumors were excluded from the study. Upon inclusion, cancer and adjacent normal tissues were excised from the included patients, which were washed with normal saline 30 min after excision, placed in nitrogen and then preserved at -80° C.

### Cell culture

Human colon cancer cell lines HT29 and SW480 were purchased from American Type Culture Collection (ATCC, Manassas, VA, USA). HT29 cells were incubated in Dulbecco's modified Eagle medium (DMEM)/F12 (1 : 1) medium containing 100 mL/L fetal bovine serum (FBS), while SW480 cells were cultured in 90% DMEM-H containing 10% FBS with 5% CO_2_ at 37° C. After reaching 80-90% confluence, the cells were detached with trypsin (Regal Biotech, Shanghai, China) and subcultured.

### Flow cytometry cell sorting

In order to isolate CD133^+^ (positive) and CD133^-^ (negative) populations, the HT-29 cells were made into single-cell suspensions, and incubated with 10 μL PBS (as a blank control), 10 μL phycoerythrin (PE)-conjugated anti-mouse immunoglobulin1 (IgG1) (as an isotype control), and 10 μL CD133/1 (AC133 clone) for 10 min under conditions void of light at 4° C. After incubation, the samples were analyzed and sorted using flow cytometry.

### Cell treatment

The cells were trypsinized and seeded into 24-well plates. The cells were then assigned into two batches of transfection below according to the instructions of lipofectamine 2000 (11668-019, Invitrogen, Carlsbad, California, USA). In the batch designed to interfere with the expression of DPP10-AS1, the cells were non-transfected or transfected with empty vector, DPP10-AS1 overexpression plasmid, si-DPP10-AS1 negative control (NC) plasmid and si-DPP10-AS1 plasmids. Meanwhile, in the batch designed to interfere the expression of miR-127-3p, the cells were non-transfected or transfected with miR-127-3p NC plasmids, miR-127-3p mimic plasmids, miR-127-3p antagomir (anta-miR-127-3p) NC plasmids, and anta-miR-127-3p plasmids. The sequences of DPP10-AS1 and miR-127-3p were obtained from the NCBI website. All the aforementioned plasmids were constructed by Shanghai Sangon Biotechnology (Shanghai, China). After 48 h of transfection, the cells were collected for subsequent experimentation.

### RNA isolation and qPCR analysis

A MiRNeasy Mini Kits (217004, QIAGEN, Hilden, Germany) were utilized to extract the total RNA content from the cells after transfection. The obtained total RNA was subsequently reversely transcribed into complementary DNA (cDNA) (10 μL) with a PrimeScript RT kit (RR036A, Takara Biotechnology Ltd., Dalian, Liaoning Province, China). Reverse transcription quantitative polymerase chain reaction (RT-qPCR) was performed using a SYBR® Premix Ex TaqTM II reagent kit (RR820A, Takara) on an ABI7500 real-time PCR instrument (Applied Biosystems, Carlsbad, CA, USA). The primer sequences of DPP10-AS1, miR-127-3p, ADCY1, CD133, CD44, Lgr5, ADLH1, U6 (as the internal control for miR-127-3p) and glyceraldehyde-3-phosphate dehydrogenase (GAPDH, as the internal control for DPP10-AS1 and other genes) were synthesized by Takara (Table 2). The 2^-ΔΔCt^ method was performed to determine the relative expression levels. Each experiment was repeated three times.

**Table 2:** Primer sequences for RT-qPCR.

**Gene**	**Primer sequences**
DPP10-AS1	F: 5'-AGGGCGTGTCTGAGATTGTG-3'
	R: 5'-TAGGAGTTCCACCGACGTGA-3'
miR-127-3p	F: 5'-TAGTTTGGAGTTAGGGGTAGGGTAT-3'
	R: 5'-AATAAATCAAAAAAAACACCTCCAC-3'
ADCY1	F: 5'-AAGGTGCCCCTACCACTTTG-3'
	R: 5'-TTTGGGAGCCGTTTCCATCA-3'
GAPDH	F: 5'-GACAGTCAGCCGCATCTTCT-3'
	R: 5'-GCGCCCAATACGACCAAATC-3'
CD133	F: 5'-CAGATGCTCCTAAGGCTTG-3'
	R: 5'-GCAAAGCATTTCCTCAGG-3'
CD44	F: 5'-CCCTGCTACCAGAGACCAAGAC-3'
	R: 5'-GCAGGTTCCTTGTCTCATCAGC-3'
Lgr5	F: 5'-CCCGGGTTTCAGAGACAACTTC-3'
	R: 5'-TCCACATGCTTTATTCCAGCAATC-3'
ALDH1	F: 5'-TCCAGCCCACAGTGTTCTCTAAT-3'
	R: 5'-GATTTGCTGCACTGGTCCAA-3'
U6	F: 5'-CTCGCTTCGGCAGCACA-3'
	R: 5'-AACGCTTCACGAATTTGCGT-3'

Note: RT-qPCR, reverse transcription quantitative polymerase chain reaction; miR-127-3p, microRNA-127-3p; ADCY1, adenylate cyclase 1 (ADCY1); GAPDH, glyceraldehyde-3-phosphate dehydrogenase; CD133, prominin 1; Lgr5, leucine rich repeat containing G protein coupled receptor 5; ALDH1, Aldehyde dehydrogenase, cytosolic; F, forward; R, reverse.

### Protein extraction and western blot analysis

Total protein content was obtained from the lysed cells using a radio-immunoprecipitation assay (RIPA) kits (R0010, Solarbio, Beijing, China) and then quantified using a bicinchoninic acid (BCA) quantitative kit (G3522-1, Guangzhou Jabes Biotechnology Co., Ltd., Guangzhou, China). Next, the protein was separated by 10% sodium dodecyl sulfate-polyacrylamide gel electrophoresis (SDS-PAGE) and transferred onto a nitrocellulose membrane. After being blocked 5% bovine serum albumin (BSA) at room temperature for 1 h, the membrane was incubated with diluted primary antibodies purchased from Abcam, Cambridge, MA, USA: ADCY1 (1 : 10000, ab203204), CD133 (1 : 1000, ab216323), CD44 (1 : 1000, ab157107), leucine-rich G protein-coupled receptor-5 (LGR5) (1 : 1000, ab75732), and aldehyde dehydrogenase isoform 1 (ALDH1) (1 : 500, ab129815) overnight at 4° C. Next, the membrane was incubated with the horseradish peroxidase (HRP)-conjugated goat anti-rabbit IgG secondary antibody (1: 5000, Beijing Zhongshan Biotechnology Co. Ltd., Beijing, China). Finally, an enhanced chemiluminescence (ECL) reagent kit was employed to visualize the results after which the gray value was calculated, with GAPDH regarded as the internal control. The cellular experiment was repeated three times to obtain the mean value.

### Dual luciferase gene reporter assay

Online biological prediction website, microRNA.org, was employed to predict the target genes of DPP10-AS1 and miR-127-3p. The targeting relationship between miR-127-3p and DPP10-AS1 as well as the relationship between ADCY1 and miR-127-3p were subsequently verified. PmirGLO Dual-Luciferase miRNA Target Expression Vector (Promega Corporation, Madison, WI, USA) was used to construct wild type (WT) pmirGLO-DPP10-AS1 vector (pmirGLO-DPP10-AS1-WT) and mutant type (MUT) pmirGLO-DPP10-AS1 (pmirGLO-DPP10-AS1-MUT). The WT vector (WT-ADCY1) and MUT vector (MUT-ADCY1) were synthesized based on the binding sequence of 3'-untranslated region (UTR) of ADCY1 mRNA to miR-127-3p. The cells were seeded in a 6-well plate (2 × 10^5^ cells/well). Next, miR-127-3p mimic and WT-DPP10-AS1 (WT-ADCY1) or MUT-DPP10-AS1 (MUT-ADCY1) were co-transfected and cultured for a period of 48 hours. Fluorescence intensity was subsequently detected using Genecopoeia's double luciferase detection kits (D0010, Solarbio, Beijing, China). Renilla luciferase was regarded as the internal reference, and the RLU value obtained from the determination of firefly luciferase was divided by the RLU value obtained from the determination of Renilla luciferase to determine the luciferase activity [35]. The experiment was repeated three times to obtain the mean value.

### RNA immunoprecipitation (RIP)

The cells were lysed in lysis buffer containing 25 mM Tris-HCl (pH = 7.4), 150 mM NaCl, 0.5% NP-40, 2 mM ethylenediaminetetraacetic acid (EDTA), 1 mM NaF, and 0.5 mM dithiothreitol supplemented with RNasin (R8060, Solarbio Life Science, Beijing, China) and protease inhibitor cocktail (B14001a, Roche Diagnostics, Indianapolis, IN, USA). The supernatant was prepared and precleared. The precleared lysate was incubated with normal IgG or anti-Ago-2 antibody (ab3238, Abcam) followed by the addition of Dynabeads G at 4° C for 4 h. The immunoprecipitated complexes were washed three times in buffer (50 mM Tris-HCl, 300 mM NaCl, pH 7.4, 1 mM MgCl_2_, and 0.1% NP-40) and eluted. Total RNA extraction was performed using the TRIzol reagent. Finally, RT-qPCR method was adopted to detect the expression patterns of DPP10-AS1 and miR-127-3p.

### RNA-pull down

DPP10-AS1, DPP10-AS1-MUT or DPP10-AS1-NC was synthesized *in vitro* by Sangon Biotech (Shanghai, China), and biotin-labeled RNA was synthesized with a T7 Megascript Kit (Thermo Fisher Scientific Inc., Waltham, MA, USA). The sample was then treated with RNase-free DNase I and purified with a RNeasy Mini Kit (Qiagen, Valencia, CA, USA) based on the manufacturer’s instructions. The whole cell lysate was incubated with the purified biotinylated transcript at room temperature for 1 h. The complex was then isolated from a streptavidin agarose bead (Sigma-Aldrich Chemical Company, St Louis, MO, USA). Finally, the RNA was purified using TRIzol, and RT-qPCR was then performed to measure the expression patterns of DPP10-AS1. The experiment was repeated three times to obtain the mean value.

### Sphere formation assay

The cells were trypsinized upon reaching 80% confluence, and subsequently prepared into cell suspensions prior to being plated in a 96 well plate (< 1000 cells/well). Six days later, when the tumor sphere grew to approximately 50 μm in size, the cell suspension was collected and resuspended to obtain purer cell spheres. The spheres were then treated with 0.25% trypsin/0.02% EDTA for 2 minutes at 37° C. Later, the spheres were centrifuged and resuspended again using CCSCs conditioned medium, followed by evaluation of sphere formation capacity. The experiment was repeated three times to obtain the mean value.

### Colony formation assay

The cells were plated in 75-mm dishes (around 800 cells per dish) and treated with samples as per the abovementioned protocols for cell treatment, and then cultured for 9 days. After undergoing methanol fixation for 20 minutes, the cells were stained with 10% Giemsa for 20 minutes, followed by counting the number of colonies (> 20 cells per dish) to calculate the rate of colony formation. The experiment was repeated three times to obtain the mean value.

### Cell counting kit-8 (CCK-8) assay

Cell suspensions (1 × 10^4^ cells/mL) were constructed with DMEM medium containing 10% FBS, which were cultured in a 96-well plate. Eight duplicated wells were set for each group (100 μL/well). At 24, 48, and 72 hours post-culture, the cells were further reacted with 10 μL CCK-8 (Sigma-Aldrich Chemical Company, St Louis, MO, USA) for 2 hours. The optical density (OD) values were recorded at 450 nm using a microplate reader (NYW-96M, Beijing Nuoyawei Company, Beijing, China). The experiment was repeated three times to obtain the mean value.

### Scratch test

The cells undergone varying transfections were inoculated in a 6-well plate. Upon reaching 90% - 100% confluence, the cells were scratched with a 10 μL pipette tip to establish a wound area (about 4 - 5 scratches were made per well). The migration distance of the leading edge of the monolayer was estimated at 0 and 24 hours. Three duplicated wells were set for each group. The experiment was repeated three times to obtain the mean value.

### Transwell assay

The cells were trypsinized after starvation for 24 hours in the serum-free medium. Subsequently, a serum-free medium Opti-MEMI (31985008, Nanjing Senberga Biotechnology Co., Ltd., Jiangsu, China) containing 10 g/L BSA was employed to resuspend the cells, with the cell density adjusted to 3 × 10^4^ cells/mL. Next, 200 μL cell suspensions were added in a 24-well plate coated with diluted Matrigel (1 : 8; 40111ES08, Yeasen Company, Shanghai, China), which was then placed into the upper layer of the Transwell chamber and allowed to solidify. Meanwhile, 600 μL Roswell Park Memorial Institute (RPMI) 1640 medium containing 20% FBS was added to the lower chamber. After incubation for 24 hours, the cells that had invaded through the membrane were fixed in 4% paraformaldehyde solution for 15 minutes and observed after staining with 0.5% crystal violet for 15 minutes. Finally, 5 visual fields were selected and the average number of the cells was determined using an inverted microscope (XDS-800D, Shanghai Caikon Optical Instrument Co. Ltd., Shanghai, China). Three duplicated wells were set for each group. The experiment was repeated three times to obtain the mean value.

### Flow cytometry

The cells were treated with 0.25% trypsin and centrifuged at 1000 rpm, and fixed with 70% chilled ethanol at 4° C overnight. The cell suspension was subsequently incubated with 10 μL RNAase at 37° C for 5 minutes and 1% propidium iodide (PI) (40710ES03, Shanghai Qcbio Science and Technologies Co., Ltd., Shanghai, China) for 30 minutes under conditions void of light. The red fluorescence was recorded at 488 nm by a flow cytometry (FACSCalibur, BD Diagnostics, Franklin Lakes, NJ, USA) to detect the cell cycle.

Meanwhile, Annexin-V-fluorescein isothiocyanate (FITC)/PI staining was performed to detect cell apoptosis following 48 h of transfection. Briefly, the cells were treated with EDTA-free trypsin, centrifugated, and reacted in an Annexin-V-fluorescein isothiocyanate (FITC)/PI apoptosis detection kit (CA1020, Solarbio, Beijing, China). The cells were then resuspended with a mixture of Annexin-V-FITC and Binding buffer (1 : 40) and incubated at room temperature. Finally, flow cytometry was carried out to measure cell apoptosis at a wavelength of 488 nm. The experiments were repeated three times to obtain the mean value.

### Xenograft in nude mice

Forty nude mice were anesthetized with diethyl ether and then inoculated with 1 × 10^6^ (200 μL) cells. Mice were subcutaneously injected via the back of the right hind leg (5 mice per group) with cells containing vector, DPP10-AS1, lentivirus-mediated small interfering RNA (siRNA) against NC, lentivirus-based siRNA against DPP10-AS1, NC mimic plasmid, miR-127-3p mimic plasmid, antagomir NC (anta-ctrl), and anta-miR-127-3p plasmid. All mice were then fed in the same environment, with the length and width of the tumors recorded every 4 days. The volume of the tumors was calculated according to the following formula: volume = length × width^2^ / 2. On the 29^th^ day, the nude mice were euthanized after which the respective tumors were dissected. Three tumors were collected from mice in each group.

### Statistical analysis

All data was analyzed using SPSS 21.0 (IBM-SPSS Inc., Armonk, NY, USA). The measurement data were expressed as mean ± standard deviation, while the enumeration data were presented as percentage or ratio. Comparisons between cancer and adjacent normal tissues were performed using paired *t*-test, while comparisons between two groups of independent samples were analyzed using unpaired *t*-test. One-way or two-way analysis of variance (ANOVA) followed by a Tukey’s post hoc test were used for the comparisons of the data among multiple groups. Data at different time points were analyzed by repeated measures ANOVA followed by a Bonferroni’s post hoc test. The correlation between data was analyzed using Pearson’s correlation coefficient. Chi-square test was used for Table 1 analysis. A value of *p* < 0.05 was considered to reflect statistical significance.
